# Assessment of Tracer ^99m^Tc(V)-DMSA Uptake as a Measure of Tumor Cell Proliferation *In Vitro*

**DOI:** 10.1371/journal.pone.0054361

**Published:** 2013-01-15

**Authors:** Fatma J. Al-Saeedi, Princy M. Mathew, Yunus A. Luqmani

**Affiliations:** 1 Nuclear Medicine Department, Faculty of Medicine, Health Sciences Center, Kuwait University, Hawally, Jabriya, Kuwait; 2 Pharmaceutical Chemistry Department, Faculty of Pharmacy, Health Sciences Center, Kuwait University, Hawally, Jabriya, Kuwait; University of Pecs Medical School, Hungary

## Abstract

**Purpose:**

To examine whether ^99m^Tc(V)-DMSA could be used as a non-invasive measure of cancer cell proliferation.

**Methods:**

Human breast cancer MCF-7, MDA-MB-231 and pII, and prostate cancer PC-3 cell lines were grown to 30, 50 and 100% confluency and pulsed with ^99m^Tc(V)-DMSA in media for 60 min at 37°C. DNA synthesis was analysed by quantification of the S phase using flow cytometry, [methyl-^3^H]thymidine incorporation and expression of proliferation markers PCNA and Ki-67 using realtime PCR. One way ANOVA was used to compare groups.

**Results:**

In all cell lines rates of ^99m^Tc(V)-DMSA uptake were inversely related to cell density. This was paralleled by similar trends in S phase proportions, [methyl-^3^H]thymidine incorporation and expression of PCNA and Ki-67.

**Conclusion:**

Rates of ^99m^Tc(V)-DMSA uptake into different types of tumour cells correlate well with cell density that is useful as a non-invasive measure of tumour cellular proliferation *in vivo*.

## Introduction

The ability to non-invasively detect and image cell growth and proliferation throughout the body has long been recognised to be of significant value in the diagnosis, staging and treatment of cancer. The expansion of a tumour mass is directly related to its growth fraction, which can be assessed by measuring DNA synthesis by following incorporation of [^3^H] or [^14^C] labelled thymidine. This is acceptable for *in vitro* model systems and in animals but as long lived beta emitters, neither radionuclide is suitable for imaging in humans. Thymidine also has the disadvantage that it is rapidly catabolised and produces large amounts of re-circulating labeled catabolites following administration [1], reducing tumour-to-normal-tissue signaling during detection and imaging of tracer incorporation into DNA by kinetic modeling [2].

Positron emission tomography (PET) has emerged as a very sensitive physiological, metabolic and molecular gamma ray detection technology that is used for imaging in many research and clinical applications but principally in oncology. For several decades the most commonly used PET tracer has been the cyclotron produced 2-[fluorine-18]Fluoro-2-deoxy-D-glucose (^18^F-FDG). However, this is neither cell specific nor very appropriate for measurement of cell proliferation, besides which it has a short half-life of less than 2 h. Several non-catabolised thymidine analogues such as ([^18^F] 3′-deoxy-3′-fluorothymidine (^18^F-FLT), have been enlisted into clinical use for lymphoma, non-small-cell lung cancer and brain tumors [3]–[6]. However, incorporation of ^18^F-FLT proceeds only to the triphosphate nucleoside step in the DNA synthetic pathway and not into DNA itself [7]. There are also some practical drawbacks of ^18^F-FLT for identifying sites of proliferative activity or malignancy in the liver and bone marrow due to the presence of high background radioactivity, and interference with pelvic lesions due to its significant excretion into the urinary bladder [8]–[11].

The short half-life of PET tracers has also limited their use to centers equipped with an on-site cyclotron. The practical need for longer-lived tracers has been resolved by the use of technetium-99m (^99m^Tc). This radiotracer, is produced by a generator that is readily available in most hospital nuclear medicine departments and does not need an on-site cyclotron to produce it. It's half-life of 6 h is long enough to perform nuclear medicine tests and short enough to minimize radiation exposure to the patient. Thus ^99m^Tc is a very useful isotope that has been incorporated into many tumor imaging agents such as ^99m^Tc-hexakis-2-methoxyisobutylisonitrile (sestamibi; ^99m^Tc-MIBI), used to detect metastatic breast cancer [12]–[13].

Pentavalent ^99m^Tc-dimercaptosuccinic acid (^99m^Tc(V)-DMSA) formed from labelling of meso-2,3-dimercaptosuccinic acid (DMSA) with ^99m^Tc under alkaline conditions, has been found to be a tumor-seeking agent. It was introduced to evaluate, image and manage medullary carcinoma of the thyroid [14]–[15]. Also it has been reported to be useful in detecting many other types of cancers such as head and neck, soft tissue tumors [16]–[17], breast [12], [18], brain [19], lung [20]–[21], bone [22], and particularly for metastatic and high-grade tumors [23] and both primary and metastatic carcinoma of the prostate [24]–[25] and melanoma [26].

Several reports from Papantoniou and colleagues [13], [27]–[29] suggest that *in vivo* ^99m^Tc(V)-DMSA uptake by breast lesions is related to proliferative activity, which is either directly related to tumor grade or to the mitotic activity.

In this study we have sought to determine whether ^99m^Tc(V)-DMSA uptake could be correlated with the rate of cell proliferation using an *in vitro* model system. Typically, attaching monolayer cultures undergo a lag phase followed by a period of rapid growth that then slows as the cells in the culturing vessel reach confluence. Confuency is the measure of % coverage of the culturing vessel. We measured ^99m^Tc(V)-DMSA uptake into several different cancer cell lines under these different conditions and compared this with assessment of other known proliferation-associated markers.

## Materials and Methods

### Materials

All general chemical reagents were purchased from Sigma (UK). Tissue culture reagents were purchased from Life Technologies (USA). Propidium iodide (PI)-ribonuclease (RNase) staining buffer (BD staining kit) was obtained from BD Biosciences (UK). Radionuclide, ^99m^Tc, was obtained from a molybdenum-99-technetium-99m (^99^Mo-^99m^Tc) generator located in the Clinical Nuclear Medicine Department of Mubarak Al Kabeer Hospital, Kuwait. [Methyl-^3^H]thymidine (specific activity 0.93 TBq/mmol) was obtained from Amersham. 2-[fluorine-18]Fluoro-2-deoxy-D-glucose (18F-FDG) was obtained from Kuwait Cancer Center (Kuwait). Dimercaptosuccinic acid (DMSA, Succimer) kit was purchased from Mallinckrodt Medical BV (Holland). Sodium bicarbonate was purchased from Pharmaceutical Solutions Industry (Saudi Arabia). Imatinib was purchased from LC Laboratories (USA).

### Cell culture and culture media

Human breast cancer MCF-7, MDA-MB-231 and prostate cancer PC-3 cell lines were purchased from Cell Lines Service (CLS, Germany). pII is an estrogen receptor down-regulated transfected cell line derived from MCF-7 [30]. All cell lines were maintained in Advanced Dulbecco's Modified Eagle's Medium (Advanced DMEM) supplemented with 5% fetal bovine serum (FBS), 10,000 units/ml penicillin, 10,000 µg/ml streptomycin, 200 mM L-glutamine and non-essential amino acids. All cells were routinely incubated in a 37°C humidified incubator in an atmosphere of 5% CO_2_-95% air. For sub-culturing, cell monolayers were harvested with trypsin-EDTA. Cell counting was carried out using a haemocytometer (Assitent, Germany) and Cell Viability Analyzer (Beckmann Coulter Vi- cell ^TM^ XR, USA). For most experiments cells were seeded into 25 cm flasks and grown to 30%, 50% and 100% confluency. All experiments were repeated thrice and individual conditions were performed in triplicate each time.

We chose breast cancer cell lines as reports from Papantoniou and colleagues [13], [27]–[29] suggest that *in vivo* ^99m^Tc(V)-DMSA uptake by particularly early breast lesions is related to proliferative activity. The evidence for this is extensively discussed in a recent review [31]. Parameters that could indicate increased proliferative activity could be very useful in predicting the progression of atypical hyperplastic or *in situ* tumours. We selected a well established and extensively used endocrine sensitive line (MCF-7) (this has also been shown to express type III NaPi co-transporters) as well as one that is naturally endocrine resistant (MDA-MB-231) and one that has acquired resistance (pII) as a consequence of siRNA transfection- the latter two being more aggressive in terms of metastatic propensity. As our aim was to determine whether our hypothesis was generally applicable, we also chose another cell line derived from a different tissue (prostate- PC-3) that has been shown to exhibit significantly higher phosphocholine as well as glycerophosphocholine levels, i.e., increased phospholipid metabolism [32]–[36].

### Preparation of ^99m^Tc(V)-DMSA

The commercial meso-2,3-dimercaptosuccinic acid (DMSA) kit (Succimer), for the preparation of trivalent technetium-99m DMSA (^99m^Tc(III)-DMSA), was used here for the preparation of technetium-99m (V) complex with DMSA. ^99m^Tc(V)-DMSA complex was prepared by addition of sodium bicarbonate solution to the kit vial followed by the addition of ^99m^Tc-eluate (Na^99m^TcO4, approximately 500 MBq). The reaction mixture reached a pH value of approximately 8 (as determined with pH indicator strips) and was then left for 15 min at room temperature. This value of pH indicates the optimum conversion to ^99m^Tc(V)-DMSA [37]–[40].

The efficiency of radiolabeling of DMSA was assessed by ascending thin-layer chromatography using Merck silica gel 60F (TLC-SG 60; Merck, Germany) and n-butanol:acetic acid:H_2_O (3∶2∶3) developing mixture. The retention factor (Rf) value for ^99m^Tc(V)-DMSA was 0.7 while for ^99m^Tc(III)-DMSA it was 0.1 giving a very clear separation [41]. The radiochemical purity was calculated as the percentage of ^99m^Tc(V)-DMSA relative to total activity on the plate. Typically we obtained efficiencies in excess of 95% conversion of ^99m^Tc(III)-DMSA to ^99m^Tc(V)-DMSA. The ^99m^Tc(V)-DMSA mixture was used within 3 h of preparation.

It has previously been reported [42] that almost all of the trivalent technetium-99m DMSA (^99m^Tc(III)-DMSA) remaining in the labeled preparation can be changed into ^99m^Tc(V)-DMSA by bubbling with pure oxygen. We therefore performed comparative uptake experiments using ^99m^Tc(V)-DMSA prepared with oxygen bubbling (100% oxygen) for 10 min or with no bubbling (0% oxygen).

The ^99m^Tc(V)-DMSA was also used for *in vivo* imaging in patients showing significant uptake into breast tumors (data not presented).

### Determination of ^99m^Tc(V)-DMSA uptake into cell cultures

All the cell lines were pulse labeled with ^99m^Tc(V)-DMSA (74 MBq/ml medium) for 60 min at 37°C. The culture medium was removed and counted to determine effluxed radioactivity using a dose calibrator (ATOMLAB 100, USA). The cell monolayers were rapidly washed three times with ice-cold phosphate buffered saline (PBS) and detached with 0.5 ml trypsin-EDTA followed by re-suspension in 5 ml of medium. The cells were centrifuged at 10,000 g for 5 min at 4°C and solubilized with 1% sodium dodecyl sulphate (SDS) in 10 mM sodium borate. The ^99m^Tc(V)-DMSA incorporated into the cellular lysate was counted as above.

Results were expressed as the total ^99m^Tc(V)-DMSA radioactivity uptake in megabecquerel (MBq) per mg of protein.

### Effect of Na+-dependent phosphate cotransporter (NaPi) inhibitors

In other experiments, MCF-7 cells were grown to about 60–70% density in a 24 well plate. These were pretreated for either 24 h or 48 h with 50 µM imatinib (prepared as a 10 mM stock solution in DMSO), or for 15 min with 5 mM and 10 mM phosphonoformic acid (PFA) prior to uptake determination of ^99m^Tc(V)-DMSA as described above.

### Extraction of radio-labeled metabolites and lipids

The intracellular fate of the ^99m^Tc(V)-DMSA (phospholipid synthesis) was determined by extraction of cell pellets with chloroform/methanol tris (hydroxymethyl) aminomethane buffer solvent system as described by Bligh and Dyer [43] and Al-Saeedi *et al* [34]. Cell pellets were re-suspended in 0.2 ml PBS in eppendorf tubes to which 0.5 ml of methanol and 0.25 ml of chloroform was added and left at 4°C for 20 min. After this period, further 0.25 ml chloroform was added to the suspension, followed by 0.5 ml 10 mM Tris buffer (pH 7) with thorough mixing. After centrifugation at 1000 g for 10 min at 4°C, the lower (lipid) phase was removed to a fresh tube and the upper (aqueous) phase was re-extracted by addition of a further 0.5 ml of chloroform, followed by centrifugation. Then both lipid and aqueous phases were counted for ^99m^Tc(V)-DMSA content.

### Fluorine-18-FluoroDeoxy Glucose (^18^F-FDG) uptake

MCF-7 cells were pulse labeled with ^18^F-FDG (13.283 MBq) for 15 min at 37°C and processed as described above for ^99m^Tc(V)-DMSA uptake.

### Effect of Na^+^ on ^99m^Tc(V)-DMSA uptake

The effect of Na^+^ on the uptake of ^99m^Tc(V)-DMSA was assessed by incubating cells for 1 h, prior to performing the assay, in two types of media in place of the standard DMEM. The high Na^+^ medium was composed of 137 mM NaCl, 5.4 mM KCl, 2.8 mM CaCl_2_, 1.2 mM Mg_2_SO_4_, 14 mM Tris (pH 7.4) and 0.1 mM KH_2_PO_4_. The Na^+^-free medium was similarly composed except that the NaCl was substituted with 137 mM N-methyl-D glucamine.

### [Methyl-^3^H]thymidine incorporation into DNA

MCF-7, MDA-MB-231, pII and PC-3 cell lines grown to different cell densities (30, 50 and 100%), were pulse labeled with [methyl-^3^H]thymidine (51.8 MBq/ml of medium per flask) for 1 h at 37°C in order to assess DNA synthesis. Cells were washed thrice with ice cold PBS, trypsinized and centrifuged at 10,000 g for 5 min at 4°C. Cell pellets were suspended with ice cold PBS and left on ice for 15 min before centrifugation. Pellets were resuspended in 1 ml of 4% trichloroacetic acid (TCA) (Fluka, UK) at 4°C and again centrifuged. Washes were repeated in 4% TCA at 4°C until the radioactivity in the supernatant was reduced to background level. To solubilise the DNA (acid-insoluble fraction), the pellet was resuspended in 0.5 ml of 4% TCA and heated to 90°C for 1 h. Then the cell debris was removed by centrifugation and washed with 0.5 ml of 4% TCA. The resulting supernatants and pellets were pooled, then suspended with Ultima Gold™ scintillation fluid (PerkinElmer, USA) and counted in a Beckman LS 6000 TA liquid scintillation counter (Beckman, USA).

### RNA extraction and measurement of gene expression

Total cellular RNA was extracted from frozen cell pellets of MCF-7, MDA-MB-231, pII and PC-3 cells using the RNeasy Plus Mini kit (Qiagen, USA) according to the manufacturer's protocol, quantitated by spectrometry and checked for integrity by standard agarose gel electrophoresis. cDNA synthesis was performed with 2 μg RNA in 20 μl using the High Capacity Reverse Transcription Kit from Applied Biosystems. Quantitative realtime polymerase chain reaction (PCR) was performed on 1 ul cDNA using a standard multiplexed Taqman PCR kit protocol (manufacturer proprietary primer/probe mixes: FAM dye-labeled TaqMan/VIC dye-labeled TaqMan (FAM/VIC) labelled target was combined with JOE dye- labeled probe, labelled normaliser actin gene oligonucleotides) to determine expression of proliferating cell nuclear antigen (PCNA), Ki67. The 20 μl reactions were performed in a 96-well plate on an Applied Biosystems 7500HT Sequence Detection System by incubation at 95°C for 10 min, followed by 40 cycles of 95°C for 15 s and 60°C for 1 min. The raw threshold cycle (CT) values were analyzed by the 2^(−ΔΔCt)^ method using the spreadsheet developed by Pfaffl [44] to determine normalized expression ratios of target genes. PCR products were also confirmed by electrophoresis on 1% agarose gel.

### Flow cytometry

For each line, cells were grown to 3 different densities in triplicate flasks to determine the S phase proportion by 2 dimensional flow cytometry analysis. For this, cells were washed twice with ice cold PBS and harvested by trypsinization. Cell pellets were fixed by adding 5 ml of ice cold 70% ethanol by constant stirring and then left overnight at – 20°C. Prior to flow cytometry, fixed cells were washed with PBS. The cell number was adjusted to 10^6^/ml. RNAse (Qiagen, USA) (1.4 µl) was added to cells which were incubated at 37°C for 15 min. This was followed by addition of 200 µl of propidium iodide (PI). Flow cytometry was performed with a Cytomics FC-500 Beckmann Coulter instrument with analysis using FC 500 Beckmann CXP software.

### Protein determination

Protein content was determined using the Bradford protein assay kit (Bio Rad, USA) after solubilisation with 1 M NaOH and neutralization with 1 M HCl. Bovine serum albumin was used as standard.

### Statistical analysis

Charting and statistics were performed using Excel and Graphpad Prismsoftware. All data are expressed as mean ± standard deviation (mean±SD) unless otherwise stated. One way analysis of variance (ANOVA) was used to determine the statistical differences between groups.

## Results

### Uptake of ^99m^Tc(V)-DMSA

**Figure 1:**
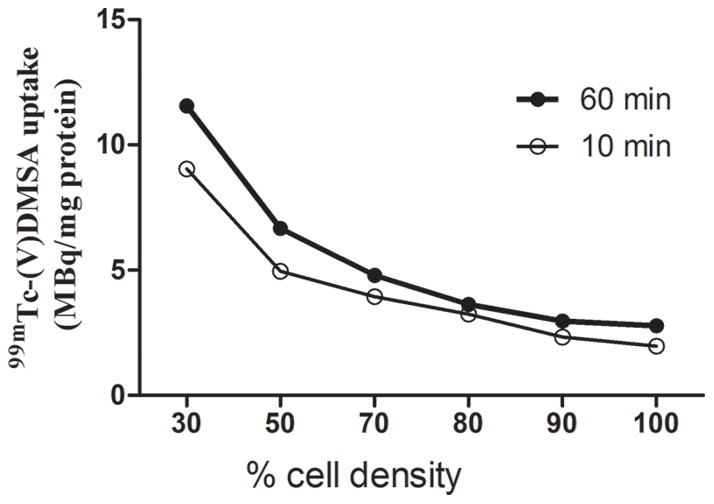
^99m^Tc(V)-DMSA uptake into MCF-7. Cells were grown to the cell densities indicated and incubated with ^99m^Tc(V)-DMSA for 10 and 60 min. Incorporated radioactivity was as described in Methods. Each point represents the mean of two samples with 5–10% variation.

Initial experiments were conducted with MCF-7 cells to optimize conditions. Cells were seeded into 25 cm flasks to achieve densities of 30, 50, 70, 80, 90 and 100% confluency. This was done by determining the number of cells needed to achieve 100% confluency after two days of growth and seeding appropriately reduced numbers of cells for the other densities. Uptake of ^99m^Tc(V)-DMSA was then measured after 10 and 60 min of incubation. The data illustrated in Fig. 1 shows that, with both time periods, the relative rate of uptake (per number of cells) was greatest at the lowest cell density and decreased with increasing cell density. For all further experiments we used incubation times of 60 min (longer times were also attempted but due to the short isotope decay time this did not offer additional advantage) and cell densities of 30, 50 and 100%. Experiments were also performed using ^99m^Tc(V)-DMSA that had been prepared with oxygen bubbling but showed no significant difference, and as this was also a technically difficult procedure to maintain sterility, its use was discontinued.

**Figure 2:**
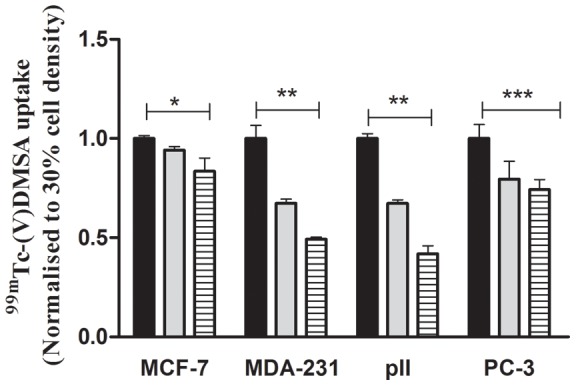
^99m^Tc(V)-DMSA uptake for each of the four cell lines used in this study. For comparison, the uptake at 30% confluency (black solid rectangular) was assigned as 1 and uptake at 50% (grey solid rectangular) and 100% (hatched bars) densities was expressed relative to this. Histobars represent mean ± SD of 3 replicates. One way ANOVA showed significant differences with p = 0.01 (*), p<0.0001 (**), p = 0.001 (***).

Fig. 2 shows ^99m^Tc(V)-DMSA uptake for each of the four cell lines used in this study. For comparison, the uptake at 30% confluency was assigned as 1 and uptake at other densities was expressed relative to this. In all cases the relative rate of uptake was inversely related to cell density. At 50 and 100% confluency, the values were 0.94±0.002 and 0.83±0.001 for MCF-7 (p = 0.01), 0.67±0.002 and 0.49±0.004 for MDA-MB-231 (p<0.0001), 0.67±0.02 and 0.41±0.07 for pII (p<0.0001) and 0.79±0.009 and 0.74±0.005 for PC-3 (p = 0.001).

The intracellular fate of the ^99m^Tc(V)-DMSA was determined in MCF-7 cells by partitioning the pelleted cellular material, following the uptake experiment, into aqueous and lipid soluble phases (phospholipid synthesis). Both phases were counted for ^99m^Tc(V)-DMSA content. Less than 5% of the label appeared in the lipid fraction indicating that almost all of the ^99m^Tc(V)-DMSA that enters the cell remains unbound to lipid during the time of the experiment.

### Uptake of [methyl-^3^H]thymidine

**Figure 3:**
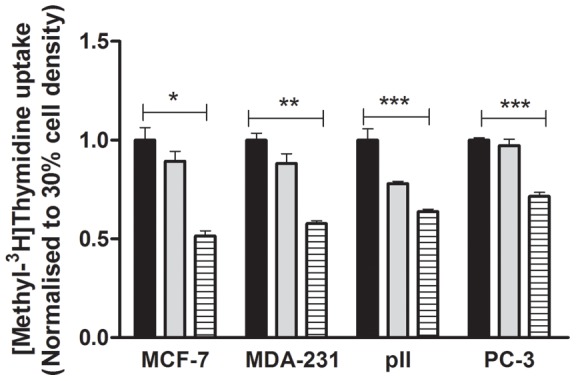
[Methyl-^3^H]thymidine uptake for each of the four cell lines used in this study. For comparison, the uptake at 30% confluency (black solid rectangular) was assigned as 1 and uptake at 50% (grey solid rectangular) and 100% (hatched bars) densities was expressed relative to this. Histobars represent mean ± SD of 3 replicates. One way ANOVA showed significant differences with p<0.0001 (*), p = 0.004 (**), p = 0.002 (***).

This was determined under the same conditions as for ^99m^Tc(V)-DMSA uptake and the results are shown in Fig. 3. With all four lines, relative uptake significantly decreased with increasing cell density.

### S-phase distribution

**Figure 4:**
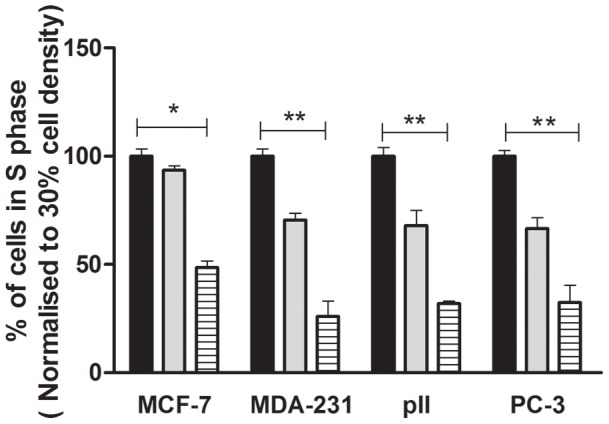
Proportion of cells in S-phase determined by flow cytometry as described in Methods at the different densities in the four cell lines indicated. Values for 50% (grey solid rectangular) and 100% (hatched bars) densities are normalized to those at 30% confluency (black solid rectangular). Each histobar represents mean ± SD for 3 replicates. One way ANOVA showed significant differences with p = 0.0001 (*) and p<0.0001 (**).

Flow cytometry was used to determine the proportion of cells in S-phase at the different densities. Significant decrease was noted as cells reached 100% confluency, in all the four cell lines (Fig. 4).

### Effect of imatinib and phosphonoformic acid on ^99m^Tc(V)-DMSA uptake

**Figure 5:**
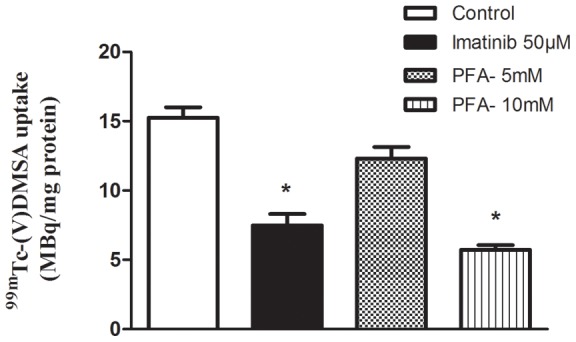
Effect of imatinib and PFA on ^99m^Tc-(V)DMSA uptake into MCF-7. Cells were grown to 60–70% confluency and then exposed to either 50 µM imatinib for 48 h or to PFA for 15 min prior to determining tracer uptake as described in Methods. Each histobar represents mean ± SD of 3 replicates. * denotes significant difference from control with p<0.001.

MCF-7 cells were exposed to either imatinib for 24 h and 48 h or to PFA for 15 min prior to measuring uptake of ^99m^Tc(V)-DMSA. Uptake was significantly decreased in the presence of imatinib by about 70% after the 24 h exposure (data not shown) and by about 60% after the 48 h exposure as compared with untreated controls, indicating that ^99m^Tc(V)-DMSA enters the cell mainly through transporters rather than simple diffusion (Fig. 5). This was further confirmed by the inhibition of uptake by PFA, a direct competitive inhibitor of NaPi cotransporters.

### Effect of cell density on expression of proliferation markers

**Figure 6:**
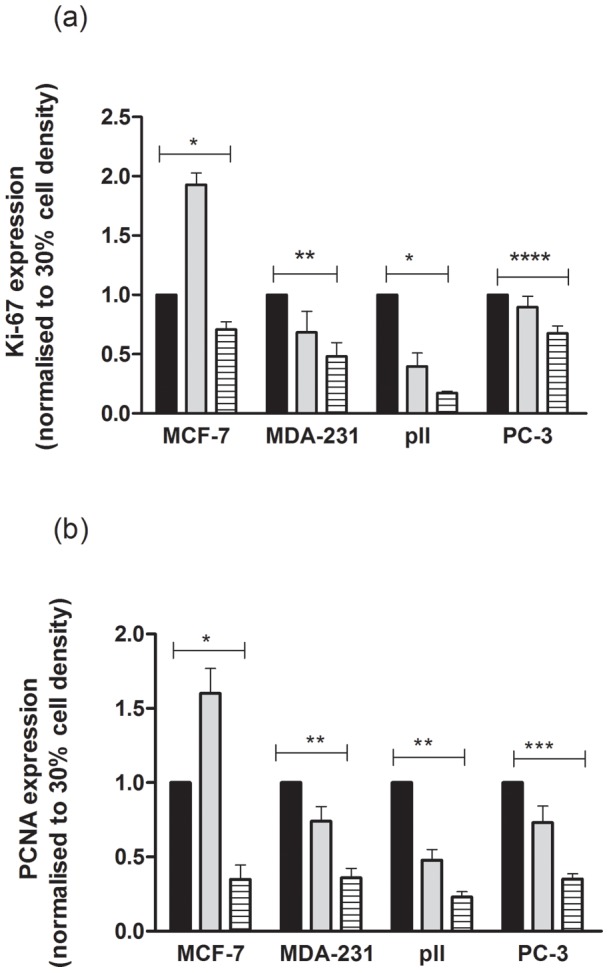
Expression of PCNA and Ki-67. RNA was extracted from cell lines grown to 30, 50 and 100% confluency as indicated, and after reverse transcription subjected to Taqman PCR as described in Methods. The 2^(−ΔΔCt)^ method was used to calculate the ratio of each gene relative to actin. Values for 50% (grey solid rectangular) and 100% (hatched bars) densities are normalized to those at 30% confluency (black solid rectangular). Each histobar represents mean ± SD for 3 replicates. (a)For Ki-67 one way ANOVA showed significant differences with p = 0.0003 (*), p = 0.006 (**) and p = 0.03 (***). (b) For PCNA one way ANOVA showed significant differences with p = 0.0008 (*), p<0.0001 (**) and p = 0.0003 (***).

PCNA and Ki-67 expression determined by Taqman quantitative PCR showed significant decrease as cells reached 100% confluency in all the four cell lines (Fig. 6). Expression in 50% confluent MCF-7 however was higher than at 30% and then decreased at 100% for both genes. This was not seen with the other cell lines.

### Fluorine-18-FluoroDeoxy Glucose (^18^F-FDG) Uptake

^18^F-FDG uptake was determined in MCF-7 cells grown to different confluencies and was found to be the same at all densities.

### Effect of extracellular Na^+^ on ^99m^Tc(V)-DMSA uptake

^99m^Tc(V)-DMSA uptake was decreased by approximately 50%, by replacing sodium with N-methyl-D-glucamine in the culture medium prior to measuring uptake.

## Discussion

In agreement with many studies reporting that ^99m^Tc(V)-DMSA prepared under alkaline conditions showed increased uptake into a variety of tumors [14 15, 17, 19, 27] we observed active uptake into several cell lines *in vitro*.

This radiotracer is not only used as a diagnostic tool but even further as a therapeutic agent. Pentavalent rhenium-188 dimercaptosuccinic acid (^188^Re(V)-DMSA) is a beta-emitting analogue of ^99m^Tc(V)-DMSA, a tracer that is taken up into a variety of tumors and bone metastases that is used on a therapeutic scale for cancer therapy [45]–[48]. For example, the treatment with ^188^Re(V)-DMSA in cases of advanced disease with osseous and soft tissue metastatic spread could be considered if these lesions are depicted by breast and whole-body ^99m^Tc(V)-DMSA scintigraphy [31]. In some reports, ^188^Re(V)-DMSA was loaded with poly(lactic-co-glycolic)acid microspheres for targeted radiotherapy and for the delivery of a radiation dose to tumors [31], [47].

The technetium is pentavalent and coordinated by an oxo-ligand and four thiolate sulfurs of two DMSA ligands. ^99m^Tc(V)-DMSA forms a small complex as [MO(DMSA)2]-, in which the M (^99m^Tc, ^188^Re) is coordinated square-pyramidically by the four thiolates and by an apical oxo-ligand and consists of mixtures of three stereo isomers of the square pyramidal mononuclear complex [49]–[50]. The negatively charged Tc(V)-oxo complex with DMSA consists of a TcO core with four sulfur atoms of the bidentate DMSA ligands arranged in a plane. In other words, the chemical structure or formula of the ^99m^Tc(V)-DMSA complex possesses four negatively charged carboxylate groups, and a central anionic technetium oxobis (dithiolato) core, i.e., [TcO (DMSA)2]^−1^ [50]–[51].

Reports as recent as 2005 and 2007 describe the use of ^99m^Tc(V)-DMSA for imaging [20], [26] and the group of Papantoniou have performed several studies on breast lesions, extensively reviewed earlier this year [31]. In this publication it is also mentioned that a protocol for use of ^99m^Tc(V)-DMSA as a guide for therapeutic administration of ^188^Re(V)-DMSA for advanced breast cancer is underway at the Vince Institute of Nuclear Sciences in Belgrade. This agent has advantages over currently employed PET radiopharmaceuticals. It is safe, cheap and readily available in many nuclear medicine centers, and can be prepared as an in house radiopharmaceutical. Unlike for example the more commonly used imaging agent ^18^F-FDG, there is no need for a cyclotron to produce it so it would be useful for medical centers that have no or limited access to such facilities for generating the current range of PET tracers. PET is not currently well-established in Kuwait; this study could provide some justification for considering a future trial to examine the utility of ^99m^Tc(V)-DMSA as a nuclear medicine marker that can be synthesized locally without the need for an expensive cyclotron.

The label was predominantly found in the aqueous fraction of cell extracts indicating actual uptake into the cell rather than immediate incorporation into membrane or other phospholipid. The comparative rates of uptake differed between the three breast and one prostate line, being higher in the more aggressive estrogen receptor negative MDA-MB-231 and pII lines. Interestingly, MCF-7 do not show invasive characteristics in *in vitro* assays [52] and PC-3 is an androgen independent prostate cell line which although displaying metastatic potential originates from a tumour type that is characteristically slower growing. In all cases however, we observed an inverse correlation between ^99m^Tc(V)-DMSA uptake and cell density. It is generally the case that the growth rate of tumors slows as they increase in size, mainly as a result of poorer vascularization and necrosis at the centre. As this is an important consideration in the design of chemotherapeutic strategies, information regarding growth rates can be clinically useful, as well indicating prognosis. Our simple *in vitro* model aims to simulate slow and faster growing groups of cells and the results suggest that the imaging tracer ^99m^Tc(V)-DMSA can provide additional information in this respect.

It is considered that ^99m^Tc(V)-DMSA utilizes the type III NaPi co-transporters [35]–[36] as phosphonoformic acid, a competitive inhibitor of NaPi co-transport, also affects ^99m^Tc(V)-DMSA uptake. Our data is consistent with studies showing that this transport is largely Na^+^ dependent.

Activated platelet-derived growth factor receptor (PDGF-R) pathway is involved in this transport mechanism. The addition of the PDGF-R inhibitor, imatinib, at least partially inhibited ^99m^Tc(V)-DMSA in our study after both 24 and 48 h of exposure to the drug, an agreement with previous studies. This drug has also been shown previously to reduce expression of these transporters [53]–[57]. Several studies reported that imatinib mesylate, a small molecule inhibiting the PDGF-R tyrosine kinase, suppressed NaPi co-transporter expression sufficiently during the time-frame of the experiment, the 24 h pre-incubation [32], [58], [59].

A recent study on U87-MG glioblastoma cells investigated an issue with obvious clinical implications, the ability of ^99m^Tc(V)-DMSA to trace the antiproliferative effects of imatinib mesylate [60]. Cells treated with imatinib for 48 h showed significant decreases in proliferation, invasion, migration and PDGF-R expression. ^99m^Tc(V)-DMSA cellular uptake studies showed that the specificaction of imatinib on PDGF-R signal pathway, in the human glioblastoma cell line U87-MG, could be followed by radioactive tracer. Furthermore, strong correlations between cellular ^99m^Tc(V)-DMSA uptake and the effect of imatinib therapy on U87-MG proliferation, invasion and migration were obtained, likewise for ^99m^Tc(V)-DMSA uptake and PDGF-R expression [60]. Besides, imatinib mesylate was initiated to treat the temozolomide-refractory tumor [55], [61].

The uptake of [methyl-^3^H]thymidine into cells and its subsequent incorporation into DNA is a common measure of cell proliferation. We have shown that its uptake into all four of our cell lines parallels that of ^99m^Tc(V)-DMSA, decreasing with increasing cell density. Similarly, flow cytometry showed that the proportion of cells in s-phase was also inversely correlated with cell density. One study has reported that ^99m^Tc(V)-DMSA brain scintitomography is a plausible non-invasive measure of glioblastoma proliferation and therapy response [58]. Recent papers have demonstrated that *in vivo* ^99m^Tc(V)-DMSA uptake has been correlated with the proliferation index measured by Ki-67 expression and phosphorylated focal adhesion kinase [19], [29], [59]. We found that expression of Ki-67 as well as of PCNA, another common proliferation marker, inversely correlated with cell density.

As a comparison, we also measured uptake of ^18^F-FDG, a commonly used imaging tracer, and found that unlike ^99m^Tc(V)-DMSA, its transport into cells was unrelated to proliferation rate, as has previously been reported [62].

In summary, we have demonstrated carrier mediated uptake of ^99m^Tc(V)-DMSA into 4 independent cancer cell lines and shown that this correlates well with proliferation rate using cells under conditions of fast and slow growth. The model has been validated with measurement of several parameters commonly accepted as markers of proliferation rate.

## Conclusion

Our data indicates that it would be worthwhile to conduct further studies to examine the extent of ^99m^Tc(V)-DMSA uptake into tumors with respect to their rates of growth so that it may have an additional use beyond detection/localization of tumours.
